# Fostering Health Literacy Responsiveness in a Remote Primary Health Care Setting: A Pilot Study

**DOI:** 10.3390/ijerph17082730

**Published:** 2020-04-16

**Authors:** Rachael Laing, Sandra C Thompson, Shandell Elmer, Rohan L Rasiah

**Affiliations:** 1 Western Australian Centre for Rural Health, School of Population and Global Health, The University of Western Australia, Karratha, WA 6714, Australia; rachael.laing@uwa.edu.au; 2 Western Australian Centre for Rural Health, School of Population and Global Health, The University of Western Australia, Geraldton, WA 6530, Australia; sandra.thompson@uwa.edu.au; 3 Centre for Global Health and Equity, Swinburne University of Technology, Hawthorn, VIC 3122, Australia; slelmer@swin.edu.au

**Keywords:** health literacy, health literacy responsiveness, environmental health literacy, primary health care, health literacy capacity building

## Abstract

Primary healthcare organisations have an important role in addressing health literacy as this is a barrier to accessing and utilising health care. Until recently, no organisational development tool operationalising health literacy in an Australian context existed. This research evaluated the efficacy of the Organisational Health Literacy Responsiveness (Org-HLR) tool and associated assessment process in a primary healthcare organisation in the Pilbara region of Western Australia. This study utilised a sequential explanatory mixed methods research design incorporating the collection and analysis of data in two phases: (1) Pre- and post-survey data and; (2) seven semi-structured interviews. Survey results showed that participants’ confidence in core health literacy concepts improved from baseline following the intervention. Analysis of the interview data revealed participants’ initial understanding of health literacy was limited, and this impeded organisational responsiveness to health literacy needs. Participants reported the workshop and tool content were relevant to their organisation; they valued involving members from all parts of the organisation and having an external facilitator to ensure the impartiality of the process. External barriers to improving their internal organisational health literacy responsiveness were identified, with participants acknowledging the management style and culture of open communication within the organisation as enablers of change. Participants identified actionable changes to improve their organisational health literacy responsiveness using the process of organisational assessment and change.

## 1. Introduction

### 1.1. Health Literacy

There has been an increase in global preventable deaths caused by lifestyle-related non-communicable diseases (NCDs), such as cardiovascular disease, type 2 diabetes and stroke, up from 57% in 1990 to 71% per year in 2016 [1]. In response, the last 20 years have seen a shift in focus away from treating acute, communicable diseases to preventing these NCDs, with increasing emphasis on self-management of health [2]. This has encouraged research to investigate effective strategies to reduce avoidable risk factors for NCDs in the population [3]. Recent efforts aimed at preventing NCDs encourage the self-management of health, whereby individuals take a more central role in managing their health status, enabling them to be partners in their own health care [4]. However, health care systems worldwide are becoming increasingly complex, increasing barriers to accessing Health care for many patients [3]. A lack of health literacy, given its crucial role in enabling patients to navigate and make use of health services and information, can be one such barrier [3].

Health literacy is a multi-dimensional and dynamic concept referring to the cognitive, personal, social, communication, and technology-use skills that enable people to navigate, access, understand, retrieve, and make use of health information in ways that promote health, thereby facilitating the self-management of health and health care decision-making [5,6,7]. Affecting populations worldwide, health literacy has been associated with the uptake of preventive services, such as cancer screening, and a range of avoidable risk factors for NCDs, such as physical inactivity, excessive alcohol consumption, and a diet high in salt, fat, and sugar [8,9]. Limited health literacy is associated with a range of poorer health outcomes, including higher rates of potentially preventable hospitalisation, increased mortality rates, and poor management of NCDs [9,10,11,12]. In addition to an individual’s skills and capabilities, studies have consistently found that social disadvantage, which is evident as low income, poor educational achievement, or being part of a cultural and linguistic minority group, are also factors that negatively affect health literacy [13,14,15].

### 1.2. Organisational Health Literacy Responsiveness

Whilst health literacy is partly determined by an individual’s personal, literary, cognitive, communicative, and technology skills and capabilities, it is also affected by the demands placed on them by their increasingly complex healthcare systems [3]. Studies have shown that primary healthcare organisations are often the first point of access into the healthcare system and thus play a central role in facilitating or impeding the health literacy skills of individuals [16,17,18]. Clinical and non-clinical staff within healthcare organisations play a vital role in alleviating health literacy as a barrier to accessing healthcare by ensuring that across their services, policies, programs, and health information are all responsive to the diverse health literacy needs and preferences of the population [19]. At a structural level, waiting rooms displaying health information that is free from medical jargon and tailored to the cultural, linguistic, and/or cognitive diversity of the population are said to be responsive to the health literacy needs of the community [20]. At an operational level, management teams that incorporate health literacy into their organisation’s policies and procedures hold their organisation accountable to being responsive to the health literacy needs and preferences of the community [21]. This is known as a healthcare organisation’s level of organisational health literacy responsiveness [15]. High standards of organisational health literacy responsiveness help facilitate equitable access to health services, particularly for those with limited health literacy, supporting preventive health and self-management of NCDs [15].

Assessing and improving organisational health literacy responsiveness is one of many continuous quality improvement (CQI) approaches undertaken in remote primary healthcare settings [22]. For example, one tool in Australia, the Audit for Best Practice in Chronic Disease quality improvement approach, has been widely popular and effective [22,23]. It includes a systems assessment tool (SAT), which enables staff in primary healthcare organisations to identify the strengths and weaknesses of their service to determine how it might be improved to ensure its effectiveness [23]. The SAT emphasizes how organisations, not just individual healthcare staff, can assist patients to self-manage their health [24,25]. Organisational health literacy responsiveness assessment emphasizes these same elements; however, the growing body of literature on CQI approaches seldom addresses it [6,21,26,27,28].

Studies have consistently found that healthcare professionals in the USA and Europe have a limited understanding of health literacy and often lack the skills required to respond to the health literacy needs and preferences of the population [29,30,31,32]. Despite this, only a small number of professional development tools and guidelines targeting organisational health literacy responsiveness have been developed [30]. The US Institute of Medicine’s (IOM) Ten Attributes of Health Literate Health Care Organisations are the most widely used internationally; however, this is not an exhaustive list and requires further development [27]. Despite having not been tested in healthcare contexts outside of the USA to date, they have been used as a framework to develop the majority of tools reported in the international and Australian literature [19,33,34]. Other tools addressing a limited conceptual scope of health literacy [33,35] are intended for use in tertiary healthcare settings only [35,36], or are for diagnostic use only, not action planning [34,36].

### 1.3. Implementing Organisational Change

Damschroder’s (2009) Consolidated Framework for Implementation Research (CFIR) highlights five key domains influencing the implementation or organisational change following an intervention (Table 1) [37].

A recent systematic review of 13 studies assessing the implementation and evaluation of organisational health literacy responsiveness initiatives internationally, including Australia, identified a number of barriers to organisational change that are consistent with the CFIR [37]. For example, a lack of culture of change and innovation and a lack of time, resources, supportive leadership, and external stakeholders all challenge the implementation of organisational change [27,38].

Published evaluations of interventions designed to improve organisational health literacy responsiveness are predominantly limited to tertiary care settings and/or metropolitan areas, so their applicability to rural healthcare contexts where NCD prevalence tends to be higher is uncertain [35,36,39,40]. Of the few studies that have been conducted in primary care settings, many are limited to community pharmacies, which represent only a limited spectrum of all primary healthcare organisations [41,42]. Thus, their applicability to a wider range of primary healthcare settings is unclear [27]. Furthermore, whilst implementation and evaluation studies have found that these tools can improve healthcare practitioners’ understanding of health literacy responsiveness [39,42], most guides have not been tested for their applicability in healthcare organisations, so their effectiveness in quality improvement approaches is unknown [27].

### 1.4. The Org-HLR Framework

**Figure 1:**
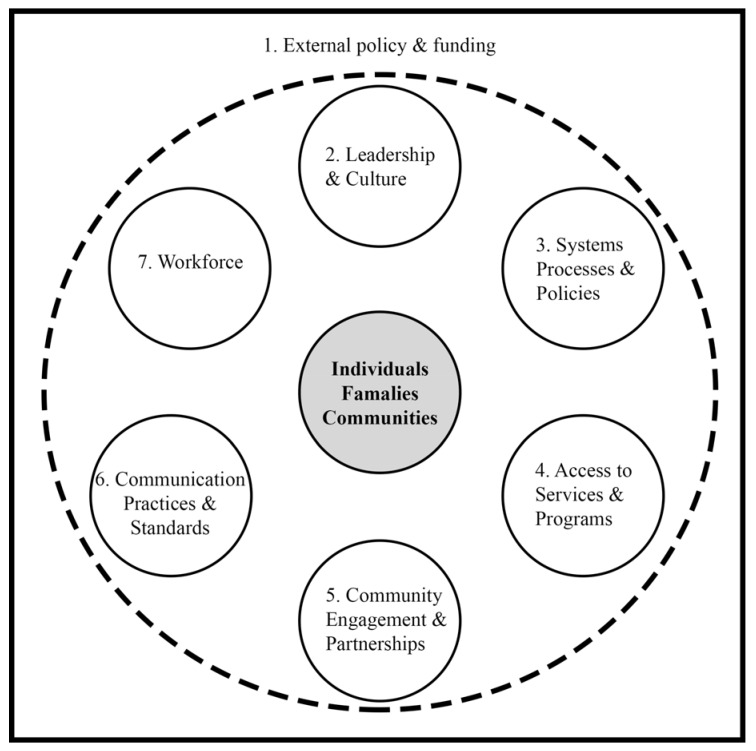
The Organisational Health Literacy Responsiveness (Org-HLR) framework domains [28]. Modified from Trezona, Dodson, and Osborne, 2017 in BMC Health Serv Res [28].

In Victoria, Australia, Trezona et al. (2017) conducted the first empirical study conceptualising organisational health literacy responsiveness in an Australian context and developed the Organisational Health Literacy Responsiveness (Org-HLR) framework (Figure 1) [28]. The framework comprises seven domains. Domain 1, the external policy and funding environment, sits outside of the direct control of organisations; however, the authors argued it is necessary to include it as a supportive policy and the funding environment facilitates an organisation’s ability to implement organisational change, which is consistent with the CFIR [37]. The Org-HLR framework formally acknowledges the influence of an organisation’s external environment [28].

Domains 2–7 of the Org-HLR framework are: Leadership and culture; systems, process, and policies; access to services and programs; community engagement and partnerships; communication and practices; and workforce, which are all interconnected and operate around a central node, individuals, families, and communities [28]. Three of these domains are additional to previous conceptualisations of organisational health literacy, more accurately reflecting the multi-dimensional nature of the concept: Systems, processes, and policies; community engagement, and partnerships; and an expanded operationalisation of the workforce domain [28].

The Org-HLR framework has guided the development of Trezona et al.’s (2018) Org-HLR Tool and Assessment Process [21]. This tool enables healthcare organisations to assess their health literacy responsiveness against the domains included in the Org-HLR framework and work collaboratively as a team to develop an action plan, addressing the gap of contextually inappropriate tools aimed at development in professionals of organisational health literacy responsiveness [21].

### 1.5. The Current Study

This pilot study implemented and evaluated the Org-HLR Tool and Assessment Process in the Pilbara region of Western Australia, classified as remote by the Modified Monash Model classification of rurality (Modified Monash Model classification: 6) [43]. Remote communities experience higher levels of social disadvantage than metropolitan areas and have poorer health literacy [2,15]. Only 12% of Pilbara residents have attained a Bachelor level qualification or higher, compared to 22% of the national population [44]. Pilbara residents are also vastly culturally and linguistically diverse as the local resource industry attracts international workers; on average, people have much lower literacy and numeracy skills, compared to the state [44,45]. Pilbara residents experience high rates of poor health outcomes compared with the state, including high rates of potentially preventable hospitalization from NCDs, such as ischemic heart disease, diabetes, and renal failure [46]. Exacerbating this, 90% of adults in the Pilbara do not consume the recommended daily servings of vegetables, and nearly 40% engage in risky alcohol consumption [46]. The substantial prevalence of social disadvantage in the Pilbara region places the community at high risk for low health literacy, which could be contributing to the high disease burden in the region [44,46].

This research explored the experiences of clinical and non-clinical staff within a primary healthcare organisation in Karratha, Western Australia, who completed the Org-HLR Tool and Assessment Process. Pre- and post-survey data were collected, and one-on-one semi-structured qualitative interviews were conducted. By using the Org-HLR Tool and Assessment Process, the participants aimed to improve the organisational health literacy responsiveness of their organisation to address the effects of limited health literacy as a barrier to accessing health care and to facilitate the self-management of health in an effort to reduce the burden of disease attributable to NCDs. The study had four research objectives:

1. To determine the baseline level of confidence in core health literacy concepts and competencies of staff within the organisation;
2. To explore the level of confidence in core health literacy concepts and competencies of staff within the organisation following implementation of the Org-HLR Tool and Assessment Process in the organisation;
3. To explore the factors affecting participants’ experiences of the Org-HLR Tool and Assessment Process; and
4. To evaluate the efficacy of the Org-HLR Tool and Assessment Process as a tool to improve organisational health literacy responsiveness within this setting.

This study adds to the current body of literature through the evaluation of an organisational self-assessment tool based on a framework that is conceptually relevant to the Australian context and facilitates the development of an action plan beyond conducting a needs assessment. This is the first study in Australia evaluating the Org-HLR Tool and Assessment Tool in a remote region.

## 2. Materials and Methods

### 2.1. Research Design

A mixed methods approach underpinned epistemologically by pragmatism and utilising survey and interview data was used. The current study utilised the sequential explanatory mixed methods design, incorporating two phases to enable the in-depth exploration of participants’ experiences [47]. Data were collected and analysed quantitatively in phase one prior to qualitative data collection and analysis in phase two. The function of using the explanatory sequential method was of complementarity, using qualitative inquiry to elaborate on quantitative data, providing a more in-depth understanding of participants’ experiences with the Org-HLR Tool and Assessment Process [14,48,49]. Ethical approval was granted by the Human Research Ethics Committee of the University of Western Australia prior to study commencement (RA/4/20/5600).

### 2.2. Sampling and Recruitment

The primary inclusion criteria for invitation to participate were that organisations were based in Karratha, Pilbara, WA and provided primary health care services to the community. This pilot study was restricted to one primary healthcare organisation, which responded to an invitation to participate in this study.

### 2.3. Participants

The organisation was a multidisciplinary not-for-profit organisation based in Karratha, WA. The organisation is comprised of a team of management, administrative, and clinical staff providing a range of primary health care services to the broader Pilbara community, including dietetics, psychology, podiatry, sonography, and chronic disease management. Both clinical and non-clinical staff were invited to participate, consistent with the CFIR [37], as facilitators of implementing organisational change that include (1) substantial buy-in from leadership; (2) a whole organisational culture of innovation and change; (3) the subject (in this case health literacy) held as an organisational priority of management; and (4) the presence of policies, procedures, and protocols that encourage health-literate practice [27]. Seven out of a total of 10 staff volunteered to participate, comprising a mix of clinicians, administrative, and managerial staff. The reason given for others not being able to participate in the workshops was their unavailability at the time due to service commitments.

### 2.4. Materials

#### 2.4.1. Health Literacy Knowledge, Skills and Experience Survey

The Health Literacy Knowledge, Skills, and Experience Survey (HLKSES) is a 14-item questionnaire measuring participants’ level of confidence in implementing a range of heath literacy competencies, developed by the Centre for Culture, Ethnicity, and Health and the HealthWest Partnership (see Supplementary Material) [50]. Items are scored on a five-point Likert scale ranging from one to five; one being the lowest, indicating *not confident at all*, and five being the highest, indicating *very confident*. Participants were required to rate their confidence on each item by circling one of the five response options on the scale for each question. From each participant’s responses on questions one to nine on the HLKSES, which focused on their individual confidence in being able to carry out a range of health literacy competencies, a mean score was calculated. From each participant’s responses on questions 10–14, which focused on their confidence in their organisation’s ability to carry out a range of health literacy competencies, a mean score was calculated. The HLKSES has not been specifically tested for its reliability and validity, however, it has been extensively used in the Australian literature, forming a major component of the evaluation of the Health Literacy Professional Development Initiatives in Victoria [50].

#### 2.4.2. The Organisational Health Literacy Responsiveness (Org-HLR) Tool and Assessment Process

The Org-HLR Tool and Assessment Process is a group-based multi-stage process consisting of three facilitated multidisciplinary workshops, each of which is accompanied by a corresponding self-assessment tool (Table 2) [21].

#### 2.4.3. Qualitative Interview Guide

The semi-structure interview guide enables flexible discussion with participants, incorporating questions pertaining to participants’: (i) Prior experience in health; (ii) understanding of health literacy and health literacy responsiveness; (iii) experiences with the Org-HLR Tool and Assessment Process workshops; (iv) reflections on the method of facilitation; and (v) perceptions on organisational change.

### 2.5. Phase 1: Quantitative

#### 2.5.1. Data Collection

Quantitative data was collected through the administration of the Health Literacy Knowledge, Skills and Experience Survey (HLKSES), completed both before and after participants completed the Org-HLR Tool and Assessment Process. Participants completed the three Org-HLR Tool and Assessment Process workshops. One workshop was completed each week over three weeks, determined by the availability of the organisation’s staff. The external facilitation was undertaken by one of the authors of the study (RR), who is external to the organisation’s staff. The facilitator is an experienced pharmacist and research with experience in facilitating group activities and was supported at the sessions by the lead author in the role of observer, assistant, and scribe.

In the first half of the first session, participants were provided with verbal and written information about the process. Following informed written consent, participants completed the HLKSES to determine their baseline understanding of and confidence in implementing crucial health literacy concepts and competencies. In the second half of the first session, participants completed the first component of the Org-HLR Tool and Assessment Process, the reflection activity, and its associated tool. During the second session, held one week later, participants completed the self-rating activity using the self-rating tool. Participants completed the third component of the Org-HLR Tool and Assessment Process, the priority-setting activity, using the priority-setting tool in a final session held one week later.

At the conclusion of the third workshop, all participants completed the HLKSES survey a second time as a post-survey, concluding the quantitative component of data collection. All participants attended at least two of the three workshops. Whilst some participants therefore had less exposure to the Org-HLR Tool and Assessment Process, pre- and post-HLKSES data were collected and analysed for all seven participants. Those who were unable to attend the third workshop were asked to complete the post-HLKSES prior to the commencement of their one-on-one interview.

#### 2.5.2. Data Analysis

The pre- and post-HLKSES data were de-identified and entered into SPSS. Following a descriptive analysis, the pre- and post-scores were paired and analysed using the non-parametric Wilcoxon signed-rank test. A separate analysis was run to track the changes in scores for the first nine items of the HLKSES, assessing participants’ level of confidence in their individual ability to carry out health literacy competencies, and the last five items, assessing participants’ level of confidence in their organisation’s ability to carry out a range of health literacy competencies. This enabled assessment of whether individuals rated a meaningful difference in their own rating on individual and organisational questions at the two time points.

### 2.6. Phase 2: Qualitative

#### 2.6.1. Data Collection

Following the completion of the quantitative phase of the study, participants were invited to participate in a one-on-one semi-structured qualitative interview. Semi-structured interviews were chosen as they allow the investigation of the research objectives in a flexible manner, addressing key research questions while allowing exploration of topics of interest as they emerge [51]. The building of trust and rapport through participant–researcher interaction is a strength of the method, enabling the exploration of more complex human experiences that would be inaccessible through quantitative methods of data collection alone [52]. Interviews lasted between 30 and 60 min each, and all were audio-recorded with informed consent [53].

#### 2.6.2. Data Analysis

Thematic analysis was used to analyse the interview data. Each interview transcript was read multiple times by the lead author (RL) to ensure full immersion in the data [54]. Coding of the data was undertaken by the highlighting of significant statements and quotes pertaining to participants’ experiences of the process. Codes with similar meanings across transcripts were then aggregated together, resulting in the formulation of themes, uncovering the experiences that were common to all participants’ transcripts. The resulting themes were reviewed by another member of the research team (RR) to verify their internal homogeneity and external distinctiveness from each other [55]. Rigour was ensured in line with Lincoln and Guba’s (1985) criteria of credibility, dependability, transferability, and confirmability [56].

## 3. Results

A total of seven staff participated in the Org-HLR Tool and Assessment Process, four of whom attended all three workshops, and three who only attended two (due to work commitments). This group included two clinicians, two administrative staff, and three managerial staff. Two of the managerial staff had clinical qualifications, and both clinical participants were early career professionals. Six participants had been in the organisation for less than six months, and only three participants had lived in the Pilbara for more than five years.

### 3.1. Quantitative Findings

The HLKSES data are described in Table 3, displaying the mean and spread of participants’ scores on the HLKSES items. To assess the level of agreement between the questions one to nine (measuring confidence in individual abilities) and the questions 10–14 (measuring confidence in organisation’s abilities) of the HLKSES, a Cronbach’s alpha score was calculated for HLKSES responses provided at both pre- and post-testing. Cronbach’s alpha values at pre-testing were 0.807 and 0.487 for questions one to nine and ten to fourteen of the HLKSES, respectively; the corresponding Cronbach’s alpha post-testing values were 0.846 and 0.795 for questions one to nine and ten to fourteen of the HLKSES, respectively.

**Figure 2:**
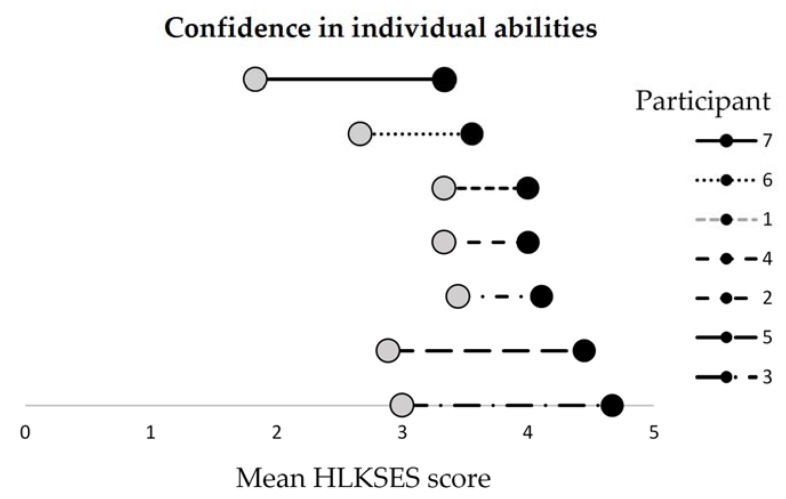
Pre- and post-survey mean scores of the Health Literacy Knowledge, Skills, and Experience Survey (HLKSES), by participant, items 1–9.

The small sample size available for quantitative analysis is acknowledged. However, analysis revealed that each participant’s mean score on the first nine items of the HLKSES increased from pre to post test (Figure 2). A Wilcoxon signed-rank test showed that participating in the Org-HLR Tool and Assessment Process led to a statistically significant increase in individuals’ confidence in their own ability to carry out a range of core health literacy competencies (*Z* = −2.384, *p* < 0.05). Indeed, the mean score rose from 2.9 at pre-test to 4.0 after the workshops were completed.

**Figure 3:**
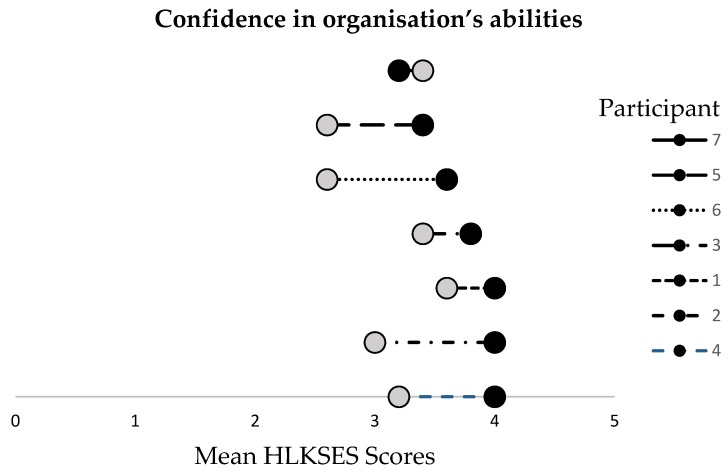
Pre- and post-survey mean scores of the Health Literacy Knowledge, Skills, and Experience Survey (HLKSES), by participant, items 10–14.

All but one participant’s mean scores on the last five items of the HLKSES increased from time one to time two (Figure 3). Participating in the Org-HLR Tool and Assessment Process led to a significant increase in participants’ confidence in their organisation’s ability to carry out a range of core health literacy competencies (*Z* = −2.209, *p* < 0.05), with the mean score rising from 3.1 (pre-test) to 3.7 (post-test).

After the workshops, participants were more confident in their own ability to carry out a range of health literacy competencies than they were in their organisation’s ability, as indicated by higher maximum mean scores on each question on the first nine questions than in the last nine questions (Figure 2 and Figure 3). The results also show that participants’ confidence in their individual abilities of health literacy increased more than for their organisation’s abilities.

One-on-one face-to-face semi-structured interviews were held. From 7 verbatim transcripts, 144 significant statements were extracted. Seven key themes were identified through qualitative analysis and triangulation of the survey and interview data. The first four themes pertain to the evaluation of the Org-HLR Tool and Assessment Process and are elaborated on in Table 4.

### 3.2. Qualitative Themes 1—4: Evaluation of the Org-HLR Tool and Assessment Process

#### 3.2.1. Theme 1: Limited Initial Understanding of Health Literacy

Participants found the Org-HLR Tool and Assessment Process workshops to be an effective educational tool, which helped them feel more confident in their understanding of health literacy. There was unanimity that prior to completing the workshops, participants’ understanding of health literacy was limited and centered around communication of health information with clients. Following the completion of the workshops, participants explained how they now understood health literacy to be a much broader concept than they had initially thought. One clinician explained how completing the workshops showed them that the relevance of health literacy extends beyond individual clients and to include the entire community.

*It’s not just the way you’re speaking to the client and what you’re discussing, it’s the other factors that are put in pace to make the service more available and to meet the needs of the community rather than the individual, so making sure the services that we’re offering are meeting the health literacy needs of the community, not just the individual.*

[Clinician 1]

Participants explained how completing the workshops highlighted that there could be a coordinated organisational response to health literacy, and that the responsibility does not lie solely with individual clinicians.

*It had never occurred to me that there could be an organisational response. It only ever seemed to me that you as a clinician or a receptionist need to understand who you’re dealing with.*

[Manager 1]

#### 3.2.2. Theme 2: Relevance of Content to the Organisation

Clinical staff found the content of the workshops to be engaging and relevant, reflecting on the value of ongoing learning, having heard of health literacy previously.

*I enjoyed it… it kind of jolted my memory… I found it really interesting.*

[Clinician 1]

Non-clinical staff enjoyed learning about health literacy, acknowledging that whilst not all of the content was directly applicable to their role, it was important to attend the workshops to gain an understanding of how their role could contribute to a coordinated organisational response to health literacy. Some participants reflected that whilst they felt engaged in the reflection and self-rating workshops, the priority-setting process in the third workshop was challenging. One clinician reflected on the difficulty of rating actions in terms of priority:

I personally found it [the third session] difficult. I found it hard to rate things with any confidence.

[Clinician 2]

Managers, on the other hand, explained that whilst consensus decision-making is a challenge, including priority setting in the workshops was an essential part of the process, as it is the most likely to lead to the implementation of organisational change and quality improvement.

*I always enjoy priority-setting, because that gives you something to focus on, to move forward. So getting, collating data is a necessary… part, but I like hearing from others what they think we should tackle first. So that was probably my, yeah, my preferred, preferred session.*

[Manager 1]

Participants made suggestions on how to improve the implementation of the Org-HLR Tool and Assessment Process, with some expressing a desire for more information about the concepts of health literacy and organisational health literacy responsiveness to be provided prior to the commencement of the first workshop, taking into consideration that the multi-disciplinary team involved included a mix of both clinical and non-clinical staff with varying background experiences with health literacy. Another participant with a clinical background commented that extra content would only be necessary if the space between workshops had been longer, reflecting the challenges of addressing different perspectives when working in a multi-disciplinary team where staff come from a range of backgrounds.

*I think how close together we did them [the workshops] was good as well, because any longer and we probably would’ve forgotten what we discussed or needed reminding.*

[Clinician 1]

#### 3.2.3. Theme 3: External Facilitation Is Important

Participants unanimously agreed that having a facilitator who was external to the organisation enabled the group discussion to be free-flowing and interactive, and indicating that perhaps if it had been facilitated internally by management, within existing formal power dynamics, this may not have been the case.

*I found the way you guys presented and facilitated was great, because there’s all that time that we could interact. But I think if [management] had been doing it, I’m not sure that we could’ve had a great amount of interaction.*

[Manager 2]

Furthermore, participants appreciated that the facilitator prompted a range of staff to provide input, increasing collaboration and open discussion.

*I thought it was quite well-facilitated asking like different people and trying to elicit their responses.*

[Clinician 2]

In addition to ensuring all members of the team shared their perspectives, participants unanimously agreed that external facilitation was important for ensuring that all topics were covered in an unbiased way, typified by one comment:

*I think it maintains the integrity of the process to have an external facilitator. Yeah. And I think the information that’s delivered is not delivered in a biased way.*

[Manager 3]

Another participant explained how having an external facilitator enabled the team to discuss their current practices with a more critical lens than they would have been able to if the sessions had been facilitated internally by management, which may have stifled critique.

*I think it was good, I think it was needed ‘cos you did sort of throw in some questions that sort of, yeah, helped us to go ‘actually no, we’re not really doing that’ or ‘yeah’ or ‘we could work on that’, so yeah as if [named the manager] ran it we might’ve just like kept going over the same things that we’re already doing, thinking, ‘oh, that’s great’, but then that sorta helped us think about other things.*

[Administrator 1]

#### 3.2.4. Theme 4: Involving the Entire Team Is Necessary

All participants commented that there was substantial value in having representatives from all parts of the organisation participate in the workshops, as this is essential to developing a common understanding across the team and to enable mutual support and collective effort towards organisational change.

*I know from experience, you can’t [just] implement things like this, it has to have an understanding across all levels.*

[Manager 1]

Another participant suggested meeting with each staff member individually for the self-rating activity before coming together to compare responses to enable them to share their opinion:

Possibly if there was time to do individual assessments, and then do the, ‘okay so, we’ve got one person that thinks it’s a 1, four people that think it’s a 5 and three in the middle’, and maybe talk about that… could be the impetus to help people speak, speak up a bit more. S-, sometimes they’ll say ‘Yep, mine’s the 1’, sometimes they won’t, but it just gives them a license to.

[Manager 1]

Despite this, it was acknowledged that the Org-HLR Tool and Assessment Process requires a substantial time commitment, particularly for clinicians with a high caseload, so some participants rejected the idea of adding more sessions to the process.

*It is already quite a long process when people are already busy… they have busy clinical schedules and might not have time.*

[Clinician 1]

### 3.3. Qualitative Themes 5–7: Implementing Organisational Change

Themes five, six, and seven relate to factors affecting the implementation of organisational change, elaborated on in Table 5. Participants across all areas of the organisation were confident that completing the process would result in organisational change. One participant explained that the team has already instigated a number of organisational changes in direct response to the implementation of the Org-HLR Tool and Assessment Process, highlighting it as an effective tool for prompting organisational change.

*It already has! We’ve already instigated different policies, um, yeah, and working around things in a different way.*

[Manager 3]

#### 3.3.1. Theme 5: Funding Limitations, Staff Turnover, and Competing Priorities

Participants did acknowledge a range of potential barriers to change. Whilst change is a current organisational priority, a large majority of the staff are relatively new to the organisation and the Pilbara itself, and are thus still in the process of navigating their roles and the region, which could reduce the human resources available within the team to implement change.

*I guess within our organisation as well because a lot of us are new or are, you know, locum or transient staff like, new to their role. I’m still new to the area, like everyone has different levels of expertise in the organisation and different focusses and still figuring out what comes under them and what comes under someone else, so it’s a bit tricky from that sense as well.*

[Clinician 1]

*The priority-setting that we did, everything that came up, I think is achievable. It’s a question of, um, time. And, um, not fiscal, human resource, so who’s best placed to do that and when are they able to physically do it.*

[Manager 1]

Alongside these internally driven challenges, participants acknowledged external drivers that could inhibit the implementation of organisational change directly resulting from the Org-HLR Tool and Assessment Process. In a regional context where there is high turnover of the health workforce, implementing organisational change that is sustainable is a challenge, as many organisations do not have staff in the region for an extended period of time. With high turnover of staff, there is a lack of consistency in how organisations are run over time, undermining the sustainability of quality improvement initiatives.

*Probably just that high staff turnover, uh, because we’ve all undertaken this process and maybe in a year’s time or two years’ time it’ll be a completely different set of staff…*

[Clinician 1]

Participants also acknowledged that limitations in funding could prohibit some changes being implemented. A sense of pessimism and lack of control related to funding was expressed.

#### 3.3.2. Theme 6: Culture of Open Communication with Management

Despite these challenges, participants appreciated a strong organisational culture valuing free open communication as an enabler of organisational change.

*I feel that… if I were to raise anything and have a discussion with [the manager, I feel would be fed up, like, it would go up to the board. I don’t feel like there’s any limitation or anything getting between me and communicating information to and from the board.*

[Clinician 1]

Another participant attested to the influence leadership has on implementing change, explaining that their senior manager is particularly receptive to discussion; however, this is not the case in every organisation, and thus implementing change could be more challenging in contexts where leadership is less open to discussion.

*I think [Senior Manager] is…different… like [they] more relatable and [they] open to discussion. Maybe if we had a different Manager who wasn’t like that, that could affect it.*

[Administrator 1]

*We’re all driven by the policy that… [named the Senior Manager] has written. But, it’s a policy that [named the Senior Manager] has written up, we’ve all reviewed [and], talked about… ‘as an organisation, this is how we’re gonna work.’*

[Manager 2]

#### 3.3.3. Theme 7: Need to Improve Induction Process

In a remote context where staff turnover rates are high, participants advocated for the incorporation of health literacy competencies into the staff induction process as a strategy to ensure that organisational change is sustainable. This was seen as particularly important given the organisation recruits staff from both clinical and non-clinical backgrounds. One participant explained that in an organisational context where staff have a range of experiences in the health sector, it is important that all entering the organisations have a common understanding of health literacy to ensure the longevity of an organisational response to health literacy.

*I think the health literacy needs to come in line with the induction process. Definitely. Has to be included so they get an understanding as well themselves because they start, especially if you haven’t had a health background, or even if you have, just still go through it to make sure you’re on the same path.*

[Administrator 2]

Management described the importance of setting standards and expectations for new staff, explaining that having a basic understanding of health literacy should be an essential criterion in the recruitment process, to ensure that the organisation’s approach to health literacy can be sustained.

## 4. Discussion

### 4.1. Evaluation of the Org-HLR Tool and Assessment Process

Participants’ confidence in both their own and their organisation’s ability to be responsive to health literacy improved from baseline following participation in the Org-HLR Tool and Assessment Process. Consistent with studies of health professionals in the USA and Europe [29,30,31,32], participants’ initial understanding of health literacy was limited to the communication of health information to individuals in a way that patients could understand. Participants described how their understanding of health literacy expanded as a result of the workshops to be a much broader concept affecting the entire community, not just individuals, in line with recent definitions of health literacy [27]. Participants highlighted that they had not considered that factors limiting community access to their services were part of health literacy. These findings support the inclusion of domain 5 of the Org-HLR Framework, community engagement and partnership, and domain 4, access to services and programs, which acknowledge the importance of extending consideration to the broader community to alleviate limited health literacy as a barrier to accessing healthcare [28].

The concept of an organisational response to health literacy was new to participants as it was previously perceived as the responsibility of individual clinicians [28]. Completing the Org-HLR Tool and Assessment Process helped participants understand that developing a coordinated organisational response to addressing limited health literacy would be a superior approach than leaving the responsibility with the clinical team. Indeed, non-clinical staff reported that completing the process showed them how health literacy related to their roles. They perceived the relative advantage of an organisational approach to health literacy over an individualised approach is consistent with Damschroder’s (2009) Consolidated Framework for Implementation Research (CFIR), which found that stakeholders’ perceptions of the advantage of implementing one intervention over an alternate solution increase the likelihood of it leading to the implementation of organisational change [37]. These findings highlight the Org-HLR Tool and Assessment Process as a preferred efficacious mechanism to enable organisational change.

The Cronbach’s alpha test results demonstrated that there was high internal consistency between the first nine questions of the HLKSES. This result is likely due to the questions being quite narrowly focussed on related aspects of the individual participants’ confidence in health literacy and understanding of how health literacy applies to their roles, both before and after the workshops (0.807 and 0.846, respectively). In contrast, the Cronbach’s alpha test scores for questions 10–14, assessing participants’ confidence in their organisation’s health literacy work practices, showed relatively little internal consistency (0.487) prior to the workshops. However, there was good internal consistency (0.795) following the workshops. Questions 10–14 address different aspects of the organisation, so the baseline inconsistency is not surprising. Throughout the workshops, it appears that the participants developed a clearer understanding of how their organisation can consistently integrate health literacy across a range of organisational work practices, accounting for the improvement in internal consistency at post-testing.

#### Factors Affecting Participants’ Experiences of the Org-HLR Tool and Assessment Process

A range of factors affected participants’ experiences of the Org-HLR Tool and Assessment Process. Firstly, its structure. Participants appreciated that given the workshops were quite long, the process was split across three separate days. This is consistent with Greenhalgh et al.’s (2004) systematic review of the implementation literature, as our study found that interventions that can be broken down into incrementally administered parts are more readily accepted [57]. However, whilst clinical participants found the time between workshops appropriate, non-clinical staff suggested that future implementation of the process would benefit from the provision of some summary content at the beginning of each workshop to ensure the content from the previous workshop was not forgotten. This reflects the challenges of implementation within a multi-disciplinary team, highlighting the importance of taking a flexible approach to ensure each participant’s needs are met.

Furthermore, whilst the aim was for all staff members of the organisation to participate in all three workshops, this was not practical in reality. To get full attendance from the entire team, it would need to be conducted outside of business hours, which has its own complications. Conducting these workshops during business hours means that essential staff will be unable to participate, as there is still a service to run and operate. Future researchers looking to implement the Org-HLR Tool and Assessment Process should take staff availability into consideration for this reason.

Participants overall found the content of the workshops engaging and beneficial. Participants’ roles affected their experience, as non-clinical staff found the content in the first two workshops more challenging to grasp than clinical staff, due to their limited backgrounds in health. Both clinical and non-clinical staff expressed that having some introductory information on health literacy prior to the first workshop would have been helpful to feel more prepared.

Participants valued that the Org-HLR Tool and Assessment Process enabled the involvement of the entire team, highlighting the importance of including clinical, non-clinical, and managerial staff. Whilst both managerial and non-managerial participants at times found it difficult to speak freely with senior managers present, they unanimously agreed that it was essential to have management present, as it enabled *all* members of the organisation to develop a common understanding of health literacy. This was seen as facilitating the implementation of an organisational response and is consistent with findings that have identified the importance of including staff from all levels within an organisation when implementing organisational health literacy quality improvement initiatives [38]. Some participants suggested holding additional breakout sessions within disciplines before coming together with management to enable people to speak more freely; however, others rejected this idea due to the time commitment required.

Participants perceived external facilitation to be an essential part of the Org-HLR Tool and Assessment Process, as it ensured the integrity of the process and reduced the impact of bias, given that senior management was present throughout. This is an interesting finding, as one of the six core characteristics that was identified from the co-design workshop underpinning the development of the Org-HLR Tool and Assessment Process was that “it can be undertaken in a timely manner, without the support of an external facilitator” [21]. This finding implies that, in contexts where managerial staff are present, organisations may gain more benefit through external facilitation rather than internal facilitation. The Org-HLR Tool and Assessment Process guidelines stipulate external facilitation as optional; thus, these findings provide important insights into contextual factors affecting successful implementation of the tool. In organisations where management will be present, our findings indicate that external facilitation should be recommended.

### 4.2. Implementing Organisational Change

Participants identified the Org-HLR Tool and Assessment Process as a useful means to improve organisational health literacy responsiveness and were confident that the process will lead to the implementation of organisational change. Participants explained that their organisational culture of innovation and ease of communication with both management and their board were key enablers of organisational change. Farmanova et al.’s (2018) systematic review also identified supportive leadership and external stakeholders as key facilitators of success in quality improvement initiatives [27], consistent with two of the Org-HLR Frameworks domains: Domain 2, leadership and culture, and domain 6, communication practices and standards [28].

Participants explored a range of internal and external barriers to implementing organisational change. Internally, participants explained that the team was in a period of growth and transition, and whilst organisational change is a current priority, there are a range of competing priorities to be achieved with limited human resources and time. Participants also acknowledged the influence of external pressures on organisational change efforts, again conscious of limitations of funding and recognising the highly transient nature of the health workforce in the Pilbara. This finding supports the inclusion of domain 1 of the Org-HLR Framework, the external funding and policy environment, as an important factor to consider that is outside the control of organisations [28]. Consistent with a range of studies in the international literature [27,38], participants highlighted that external factors could not be ignored when implementing organisational change.

Participants identified the Org-HLR Tool and Assessment Process as an effective tool for highlighting the importance of succession planning, specifically, by including health literacy in the staff induction process, in line with domain 3 of the Org-HLR Framework, systems, policies, and processes. Participants unanimously acknowledged that incorporating health literacy into the induction process for all staff, including clinical, administrative, and managerial staff, would help alleviate the impact of the highly transient workforce and agreed that focusing on workforce development would have a positive influence on the sustainability of organisational change.

### 4.3. Limitations and Future Directions

This was a pilot to inform a larger study evaluating the Org-HLR Tool and Assessment Process in the Pilbara. Whilst the results of the study provide valuable information that can guide future implementation of the tool (Table 6), there were limitations. Firstly, the lack of evidence in the published literature describing the reliability and validity of the HLKSES is a limitation, but the tool provides an indication of the change in confidence of the participants’ understanding of health literacy and its application. Secondly, only one organisation was selected for this pilot, and not all employees were able to participate due to the substantial time commitment required to complete the workshops. This meant that clinical employees with higher caseloads were unable to participate, undermining representation from the entire clinical team. The study was also limited due to the small sample size available for quantitative analysis. Despite these limitations, the seven staff who participated reflected the multi-disciplinary make-up of the organisation, including participants across its management, administration, and clinical teams.

The evidence would be strengthened by further studies implementing and evaluating the Org-HLR Tool and Assessment Process in additional primary healthcare organisations in the Pilbara and collecting quantitative data from a larger number of participants from a range of organisations. Future research would also benefit from multiple case studies to explore the applicability of this study’s results to a range of healthcare settings in remote Australia, as it is unlikely that the intervention will impact all remote healthcare organisations in the same way. Furthermore, whilst the participants included in this pilot study represented a broad range of healthcare roles, the two clinicians were both professionals with only a few years of clinical experience. Thus, the results of this study cannot be generalised to experienced clinicians in primary healthcare. More research is required to understand the health literacy knowledge of experienced clinicians.

## 5. Conclusions

This pilot was the first study to evaluate the Org-HLR Tool and Assessment Process in remote Australia. The results from the quantitative and qualitative analyses were integrated into an essential schema of participants’ experiences with the Org-HLR Tool and Assessment Process. Two broad categories of themes were identified, with four pertaining to the evaluation of the tool and assessment process itself, and three related to the organisation’s perception of and confidence in the implementation of organisational change.

The Org-HLR workshops were educationally effective, with participants learning that health literacy is a much broader concept than they initially thought, encompassing (1) a consideration of the needs of the entire community, not just communication of health information in to individuals, and (2) one that can have an organisational response requiring commitment and action from both clinical and non-clinical staff members, not just individual clinicians. Participants found the content of the workshops relevant and necessary, even if not every single piece of information was relevant to each person’s individual role.

Given the length of the process, participants appreciated that the sessions were manageable by being delivered as three workshops not spread too far apart. It was echoed across the team that whilst some initially felt unsure to speak up in the presence of management, it was important in forming an organisational response to have the entire team involved. External facilitation was seen as protecting the integrity of the process and reducing potential bias that may have occurred with internal facilitation. Recommended improvements included the provision of more information about health literacy before each workshop. Some participants expressed a desire for sessions where they participants could speak freely and frankly prior to group workshops and group decision-making.

Participants were confident that engaging in the process will lead to organisational change in the context of an organisational culture valuing open communication with management, albeit recognising external barriers present, which include funding limitations, high staff turnover, and a range of competing priorities being present in the organisation’s current configuration. Finally, participants unanimously agreed that one of the most pressing issues to address that emerged from workshop discussions was the need to improve the induction process.

The results of this evaluation add to the organisational health literacy responsiveness literature in Australia, as the Org-HLR Tool and Assessment Process is based on the Org-HLR Framework, which is the first framework operationalising organisational health literacy for use in the Australian context. Further evaluations need to be conducted to determine the applicability of the Org-HLR Tool and Assessment Process in a range of remote contexts within Australia. Whilst this pilot study involved a single primary healthcare organisation in Karratha, Western Australia, the information gained from the evaluation indicates that the Org-HLR Tool and Assessment Process could be usefully adapted for further use in the Pilbara and similar remote health services in Australia. These adaptations need to be considered for future use of the Org-HLR Tool and Assessment Process. This study has demonstrated the potential of organisational health literacy responsiveness, which is important for improving the self-management of NCDs at the local health service level and can help efforts to reduce the disease burden.

## Figures and Tables

**Table 1:** The Consolidated Framework for Implementation Research domains [37].

No.	Domain
1.	Outer setting
2.	Inner setting
3.	Individuals involved
4.	The intervention itself
5.	The implementation process

**Table 2:** The Three Organisational Health Literacy Responsiveness (Org-HLR) workshops [21].

Workshop	Purpose	Tool Used	Time Required
Reflection activity	To encourage reflection and discussion about health literacy concepts, the specific health literacy needs of clients and communities, and the organisation’s role in responding to them	Reflection tool	60–90 min
Self-rating activity	To enable the organisation to assess their health literacy responsiveness against a set of performance criteria, allowing the identification of strengths and weaknesses in organisational capacity and performance	Self-rating tool	3–4 h
Priority-setting activity	To support the organisation to prioritise actions and improvement activities based on areas of weakness identified previously	Priority-setting tool	2–3 h

**Table 3:** Descriptive statistics of the Health Literacy Knowledge, Skills, and Experience Survey (HLKSES) results.

	N	Mean	SD	Min	Max	Median
**Q 1–9**						
Pre	7	2.9	0.56	1.8	3.4	3.0
Post	7	4.0	0.46	3.3	4.7	4.0
**Q 10–14**						
Pre	7	3.1	0.40	2.6	3.6	3.2
Post	7	3.7	0.32	3.2	4.0	3.8

**Table 4:** Key themes identified relating to the evaluation of Org-HLR Tool and Assessment Process.

Theme.	Elaboration
Understanding of health literacy	All participants explained that their understanding of health literacy had broadened now that they have completed the Org-HLR Tool and Assessment Process.
Relevance of content	Overall, participants found the content relevant to their roles, and there was a consensus that the process would benefit from the inclusion of more content at the beginning explaining the concepts of health literacy and health literacy responsiveness to ensure all team members had a shared understanding.
External facilitation	Having an external facilitator protected the integrity of the process and reduced bias, ensuring that all topics were critically explored across the team.
Entire team involvement	Including staff across the organisation, including from management, administrative, and clinical roles, was an important part of planning for organisational response to health literacy, despite acknowledgement that having management in the room can make some feel less confident to speak.

**Table 5:** Key themes identified relating to implementing organisational change.

Theme	Elaboration
Barriers to change	High staff turnover, funding limitations and competing priorities were identified as potential external barriers to implementing organisational change.
Enablers of change	A strong culture of open communication with management which made discussing change easier was identified by respondents across the organisational team.
Induction process	Participants agreed that the organisation’s induction process needs to improve, including incorporating health literacy concepts for all staff, both those in clinical and non-clinical roles, to ensure everyone commences their role with a baseline understanding of health literacy.

**Table 6:** Key recommendations for future use of the Org-HLR Tool and Assessment Process in the Pilbara.

No.	Key Recommendations
1.	A brief review of health literacy and organisational health literacy responsiveness should be provided at the beginning of each workshop in organisations where non-clinical staff are involved.
2.	Additional break-out sessions within team should be offered to organisations where time permits, before bringing all staff together for group decision-making.
3.	Managerial, clinical and non-clinical staff should be encouraged to participate in the process.
4.	External facilitation should be recommended where management is included in the workshops.
5.	Implement and evaluate the Org-HLR Tool and Assessment Process in further primary healthcare organisations in the region.

## Supplementary Materials

The following are available online at https://www.mdpi.com/1660-4601/17/8/2730/s1. File S1. Health Literacy Knowledge, Skills and Experience Survey.
