# Supplementary material for: Fostering Health Literacy Responsiveness in a Remote Primary Health Care Setting: A Pilot Study
Source: Int J Environ Res Public Health. 2020 Apr 16;17(8):2730. doi: 10.3390/ijerph17082730 (PMC7215686; doi:10.3390/ijerph17082730)
Supplement: Supplementary file 1 [file ijerph-17-02730-s001.pdf]

Supplementary Materials

S1.Health Literacy Knowledge, Skills and Experience Survey

On a scale from 1 to 5 (1 is the lowest, not confident at all; 5 is highest, very confident), please indicate your **level of confidence** in being able to do the following:

Health Literacy Competencies

1. Explain differences in the various ways that health literacy is defined and conceptualised
   1 2 3 4 5
2. Outline patient indicators and outcomes of low health literacy
   1 2 3 4 5
3. Explain that it is the responsibility of the health sector to address the mismatch between patients' and health care providers' communication skills and tactics
   1 2 3 4 5
4. Explain the relationship between health literacy and health equity / Identify population groups that are at increased risk of low health literacy
   1 2 3 4 5
5. Outline the risk management and quality improvement imperatives of improved health literacy
   1 2 3 4 5
6. Describe the rationale for applying a universal precautions approach to health literacy
   1 2 3 4 5
7. Recognise, avoid and/or constructively correct the use of medical jargon
   1 2 3 4 5
8. Effectively use the Teach-Back technique for assessing patients' understanding
   1 2 3 4 5
9. Apply plain language principles in written communication and write in English at the grade 5 level
   1 2 3 4 5

On a scale from 1 to 5 (1 is the lowest, not confident at all; 5 is highest, very confident), please indicate your **level of confidence** in being able to do the following:

My organisation is

10. Integrating health literacy into planning, evaluation measures, patient safety, and quality improvement
    1 2 3 4 5
11. Preparing the workforce to be health literate and monitors progress
    1 2 3 4 5
12. Including populations served in the design, implementation, and evaluation of health information and services
    1 2 3 4 5
13. Meeting the needs of populations with a range of health literacy skills while avoiding stigmatisation
    1 2 3 4 5
14. Using health literacy strategies in interpersonal communications and confirms understanding at all points of contact
    1 2 3 4 5

References

1. Naccarella, L. Evaluation of 2014 Health Literacy Professional Development Initiatives; The University of Melbourne, Melbourne, Australia, 2015; pp. 1–58.
